# Impact of UV-B Photoaging on Chlorpyrifos Adsorption by PET Microplastics: Insights from Experimental and DFT Analysis

**DOI:** 10.1021/acsomega.4c07521

**Published:** 2024-11-06

**Authors:** Thais B. O. Costa, Giuliana B. Santana, Eric M. Silva, Kelven G. A. Conceição, Gabriela Z. Diaz, Diego Q. Melo, Antonia Mayza M. França, Ronaldo F. Do Nascimento, André G. Oliveira, Rílvia S. Santiago-Aguiar, Othon S. Campos, Carla B. Vidal

**Affiliations:** † Department of Chemistry and Biology, Federal University of Technology − Paraná, Deputado Heitor de Alencar Furtado St., Five Thousand Ecoville, Curitiba, PR 81280-340, Brazil; ‡ Federal Institute of Education and Science of Sertão Pernambucano, PE 647, km 22, PISNC N-4, Campus Petrolina Zona Rural, Petrolina, PE 56302-970, Brazil; § (Actual) Department of Analytical Chemistry and Physical Chemistry, Federal University of Ceará, Humberto Monte S/N Campus do Pici, Bloco 940, Fortaleza, CE 60451-970, Brazil; ∥ Center of Technological Sciences, University of Fortaleza, Av. Washington Soares, 1321, Edson Queiroz, Fortaleza, CE 60881-905, Brazil; ⊥ Department of Chemical Engineering, Federal University of Ceará, Campus do Pici, Bloco 709, Fortaleza, Ceará 60455-760, Brazil; # Department of Physics and Chemistry, Federal University of Espirito Santo, Alto Universitário SN, Guararema, Alegre, ES 29500-000, Brazil

## Abstract

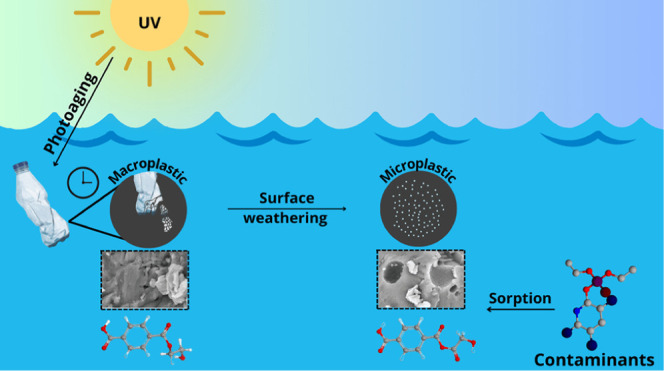

Microplastics (MPs) in the environment pose a persistent concern, as these plastic particles can adversely impact both aquatic ecosystems and human health. MPs are subject to natural weathering and aging processes, such as photodegradation, which significantly alter their surface morphology and physicochemical properties, thereby influencing their fate, transport, and sorption behavior. To better understand how aging affects these properties and to elucidate the mechanisms behind the interactions with the chlorpyrifos pesticide, poly(ethylene terephthalate) (PET) microplastics (<100 μm) were subjected to accelerated photoaging in a UV-B chamber for varying exposure times. Several characterization techniques were employed, including scanning electron microscopy (SEM), energy dispersive X-ray spectrometry (EDS), X-ray diffraction (XRD), infrared spectroscopy (IR), point of zero charge (pHPZC), and thermogravimetric analysis (TGA). Additionally, the percentage of crystallinity and carbonyl index (CI) were calculated. The results demonstrated a decrease in pHPZC following photodegradation, likely due to an increase in δ- (−C=O) groups, consistent with the CI findings. SEM revealed surface deterioration caused by aging, while TGA indicated an early thermal degradation stage associated with water volatilization, suggesting enhanced hydrophilicity. Density functional theory (DFT) calculations showed that the PET monomer absorbs slightly in the UV-B region, leading to excitation and subsequent radical reactions that form Norrish type I products. The adsorption capacity of aged PET increased compared to the pristine material, likely due to the molecular geometry becoming more planar, facilitating interactions between the aged MPs and chlorpyrifos, as confirmed by noncovalent interaction (NCI) analysis. These findings highlight the significant potential of aged microplastics to adsorb micropollutants and act as vectors in aquatic environments.

## 1 Introduction

According to the Organisation for Economic Co-operation and Development (OECD), global plastics production has grown significantly, raising awareness about plastic pollution and driving public opinion toward more decisive policy interventions.^1^ Approximately 60% of all plastics produced have been discarded and accumulated in landfills or the natural environment and can be broken into microplastics (MPs) (plastic particle sizes ranging from 1 to 1000 μm).^2,3^

PET is one of the most widely used plastics globally, commonly found in products such as bottles, films, tarpaulins, canoes, liquid crystal displays, holograms, filters, dielectric films for capacitors, wire insulation, and insulating tapes. While PET is technically and economically more recyclable than many other plastics and despite the existence of a well-established recycling infrastructure, it remains one of the most frequently detected microplastics in marine environments. This prevalence is why PET was chosen as the focus of this study.^1,4−6^

Due to MP properties=such as crystallinity, particle size, large specific surface area, hydrophobicity, and surface functional groups=microplastics (MPs) pose a significant environmental threat. These pollutants can adsorb toxic compounds from their surroundings, acting as anthropogenic vectors that contribute to ecotoxicological effects on aquatic organisms and facilitate the transfer of contaminants through the food chain.^7−9^

In addition, the MPs in the environment undergo natural weathering and aging processes=such as physical abrasion, heat, UV irradiation, chemical oxidation, microbial attachment, and biodegradation=which can significantly affect their surface morphology and physicochemical properties. These changes may affect their fate, transport, and sorption behavior. Therefore, it is crucial to investigate the role of photoaging in the adsorption mechanisms of pollutants commonly detected on MPs.^3,10^ As an alternative to natural processes, some studies highlighted the use of accelerated aging chambers, which may provide insights into the long-term aging behavior of MPs through artificial control of UV intensity.^3,11,12^

On top of that, the environmental contamination caused by pesticides and their harmful effects on the health of aquatic ecosystems are also a big issue. Several studies report the occurrence and persistence of many pesticides in the marine environment.^13,14^ Chlorpyrifos (CP) is a hydrophobic organic pesticide of the organophosphorus class, whose mechanism of action is to inhibit the nerve transmissions of insects until their nervous system collapses; being one of the most commercialized active ingredients in Brazil for application in corn, soybean, and cotton crops.^15^

In this context, quantum mechanics methods such as density functional theory (DFT) have become indispensable tools for laboratory experiments in the modern world. These calculations bring new insights into typical results that can deepen the system knowledge, providing results that cannot be acquired by ordinary laboratories or equipment, since the DFT is a methodology for solving the Schrödinger equation for many-body systems, which can be translated as a molecule. Although this topic has difficulties, modern computer systems can help one obtain those results relatively quickly, making it possible to obtain spectroscopy data, thermodynamic values, and so on.^16^

Since microplastics and pesticides are widely detected in the aquatic environment, the adsorption of pesticides by MPs may result in more significant toxicity to marine organisms. Based on the above, this paper aims to investigate the effect of accelerated photoaging on poly(ethylene terephthalate) (PET) microplastics under different aging days on physicochemical and morphological properties and chlorpyrifos sorption behavior.

## 2 Materials and Methods

### 2.1 Chemicals and Materials

Micronized PET samples (<100 μm in diameter) were purchased from the MICROPET in Ribeirão Pires-SP, Brazil. A high purity standard of chlorpyrifos (CP) (≥98.0%, CAS #2921-88-2) was purchased from Sigma-Aldrich, USA. HPLC-grade acetonitrile (ACN) and methanol (MeOH) were obtained from Merck.

The stock solution of CP was prepared in methanol at 100 mg L^–1^, and the working solutions were prepared by diluting appropriate aliquots of the stock solution in water.

### 2.2 Accelerated Aging of PET

UV irradiation can be employed to simulate the damaging effect of sunlight on materials such as aged microplastics. In detail, the PET microplastics (10 g) were evenly dispersed in quartz Petri dishes, which were positioned 30 cm from the lamps and exposed to UV light (8 × 40 W UV-B bulbs, 315–280 nm) in an accelerated aging chamber provided by the company COMEXIM (São Paulo, Brazil) (Figure S1) with controlled temperature at 60 °C at different exposure times (1, 4, 6, and 8 h), following the ASTM^17^ test method. After aging, PET microplastics were stored in clean storage bags for further use, and the samples were named PET (pristine), PET 1h, PET 4h, PET 6h, and PET 8h (Figure S2).

### 2.3 Characterization Method

Scanning electron microscopy (SEM) images were obtained with the gold coating technique in an EVO MA 15 (Zeiss) microscope by using the secondary electron signal in a high vacuum environment, with an acceleration voltage set at 20 kV, a work distance of 7 mm, and magnifications of 2,000 and 5,000 times. The samples were placed on carbon tape and covered with a thin layer of gold by using a Q150R ES quorum metallizer. The sputtering process lasted 45 s, with a sputter current 30 mA, resulting in a coating thickness of 7 nm.

Fourier-transform infrared spectroscopy (Shimadzu IR Tracer-100 spectrometer, Japan) operating in the 4000–400 cm^–1^ range, with a nominal resolution of 4 cm^–1^ and an accumulation of 64 scans, prepared in KBr (3 wt %), was employed to assess the alteration in the surface functional groups of both pristine and aged microplastics.

The areas of the bands referring to the carbonyl groups (C=O) and symmetric grouping (CH_2_) were used to calculate the carbonyl index (CI), as expressed in eq 1:^18^
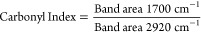
1

The crystallinity of microplastics was analyzed using X-ray diffraction (Shimadzu Co model XRD-7000, Japan). The standards were collected in continuous mode with a 2θ scan speed of 2° min^–1^, with the tube operating at 30 kV and 30 mA. Amorphous and crystalline areas were used to calculate the materials’ crystallinity percentage, as established by eq 2.^18^
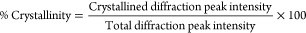
2

Thermogravimetric analysis (TGA) was performed on approximately 10 mg of the sample, placed in an aluminum pan, using a TGA Q50 instrument (TA Instruments, Delaware, USA). The analysis was conducted in a synthetic air atmosphere with a flow rate of 60 mL/min and a heating rate of 10 °C/min over a temperature range from 25 to 900 °C.

Determination of pH_PZC_ of adsorbent materials was carried out using the method described by Herath et al.^19^ and Hlekelele et al.^20^ The procedure involved mixing 1.0 g of each material with 25 mL of 0.1 mol L^–1^ NaCl solution under 11 different initial pH conditions (varying from 2 to 12). pH was measured before and after 24 h of shaking on an orbital table at 150 rpm at 25 ± 2 °C, using an Even pH meter (model PHS-3E) and a SOLAB orbital shaker (model SL222). The results were expressed through the ΔpH (pHi – pHf) graph versus pH_i_, in which the pH of pH_PZC_ was determined.

### 2.4 Batch Adsorption Experiment

The effects of PET microplastic photoaging and pristine on chlorpyrifos’ adsorption were determined via batch adsorption tests. Microplastics were placed in 125 mL glass flasks, each containing 50 mL of a synthetic chlorpyrifos solution at a concentration of 1 mg L^–1^ and at an MP dosage of 0.2 mg mL^–1^. The glass flasks were agitated in a rotating incubator at a temperature of 30 °C, with a pH set at 7.0, for 24 h at 170 rpm.

The adsorption tests were conducted in triplicate, and only the mean values were reported. The samples were filtered through 0.25 μm PTFE filters, and the residual chlorpyrifos concentrations were detected using a liquid chromatograph (model Agilent 1260 Infinity) equipped with a diode array detector (DAD G4212B), using a C18 Hichrom5 column (25 cm × 4.6 mm I.D., 5 μm). Analysis followed an isocratic mobile phase (20:80) of acetonitrile/water (ACN/H_2_O). The flow rate was 0.8 mL min^–1^, the oven temperature was maintained at 35 °C, the injection volume was set at 20 μL, and the detection was at 230 nm.

The adsorption capacity (*Q*) was calculated as follows: eq 3:
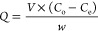
3

Where *Q* (mg g^–1^) is the adsorption capacity, *V* (L) is the volume of the synthetic solution, *C*_o_ (mg L^–1^) is the initial concentration of the pesticide, *C*_e_ (mg L^–1^) is the equilibrium concentration, and *w* (g) is the mass of PET microplastics.

Analysis of variance (ANOVA) was used to test the hypothesis for mean comparisons across the groups. When ANOVA indicated a statistically significant difference between group means, the Student’s *t* test was applied to compare the means of two specific groups.

### 2.5 Quantum Chemical Calculations

All quantum chemical calculations were conducted in an AMD-type computer with optimized hardware running in the Windows 11 environment. Both oxidized and integer PET molecules were drawn using Avogadro 1.20 software,^21^ and the structural file was optimized using the GFN-XTB package.^22^ For DFT calculation, the ORCA 5.0.4 package was used,^23^ and a PBE0-D3 functional with dispersion correction^24^ along with Ahlrichs def2-TZVP correlated basis set, excluding *f* orbitals^25,26^ was used. This configuration was used for obtaining the UV–vis spectrum by applying the TD-DFT method. For obtaining the orbital data into readable files, the orca plot tool converted the files into .cube files for treatment in Gabedit 2.5.1^27^ for obtaining UV–vis and Chemcraft 1.8^28^ and IboView v20211019-RevA^29^ for obtaining and rendering the calculated orbitals, respectively. The calculated noncovalent interaction (NCI) was conducted using MultiWFN version 3.8.^30^

## 3 Results and Discussion

**Figure 1:**
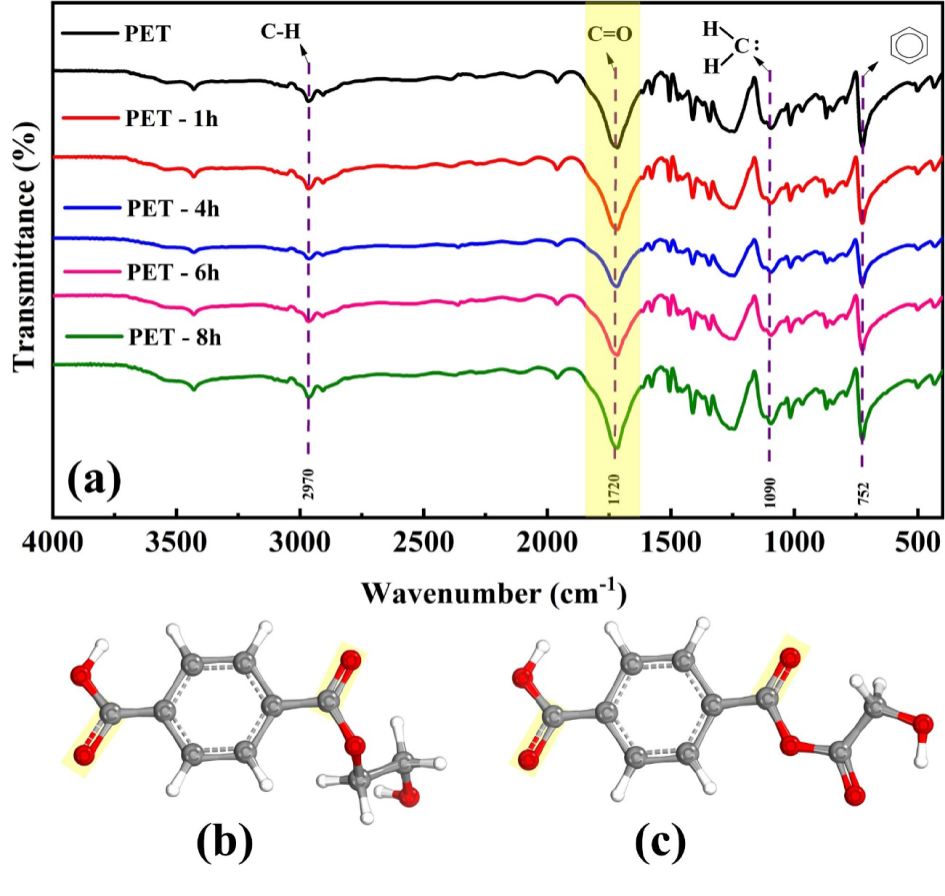
FTIR spectra of PET samples before and after undergoing accelerated aging (a) and molecular models of PET monomer before (b) and after UV-B aging (c).

Figure 1a presents the infrared spectra for the microplastic samples. The main absorption bands in the FTIR spectrum of PET were identified as follows: bands in the range of 3100–2800 cm^–1^ are associated with the stretching of aromatic and aliphatic −CH– bonds. Furthermore, the bands at 1300 and 1100 cm^–1^ are attributed to the stretching of the methylene group linked to the oxygen of the ester, while the band at 1090 cm^–1^ refers to the methylene group, and the bands at 1016 and 725 cm^–1^ are associated with aromatics. Many medium and weak intensity bands are attributed to polymer chain configurations,^31,32^ while the band at 1720 cm^–1^ corresponds to the stretching of the carbonyl (C=O) group in the ester bond.

This last band was expected, as PET already contains this functional group in its chemical structure. However, an increase in the C=O intensity was observed in the photoaged materials, particularly in PET-8h, suggesting possible oxidation processes following UV-B irradiation, which is consistent with findings from previous studies.^18,33,34^

This can be attributed to the Norrish type I reaction (Figure S3). In this process, oxygen bonded to the ester group becomes a radical. An oxygen molecule then interacts with the radical carbon, forming a bond, while a neighboring polymer molecule donates a hydrogen atom to stabilize the radical. The resulting peroxyl group undergoes dehydration, forming a new C=O bond in the aged PET molecule, leading to a geometrical change: since the C=O bond is of sp^2^ type, the aged molecule becomes more planar compared to the pristine PET molecule (Pitts and Blacet, 1950),^200^ illustrated in Figure 1b,c.

PET’s aging process occurs in the UV-B region, reflected by a small absorption peak at 288 nm in the calculated spectra (Figure S4). This peak corresponds to the HOMO+3 excited state, where electrons are promoted to the third energy level above the ground state of the PET monomer. This energy is sufficient to trigger the Norrish type I reaction at the C–H bond, consistent with the C–H bond energy (414 kJ mol^–1^ or 289 nm, as per the Planck equation) (Jones, 2016).^201^ The calculated energy gaps between HOMO–LUMO and HOMO+3–HOMO are 4.65 and 0.83 eV, respectively (Figure S4).

**Figure 2:**
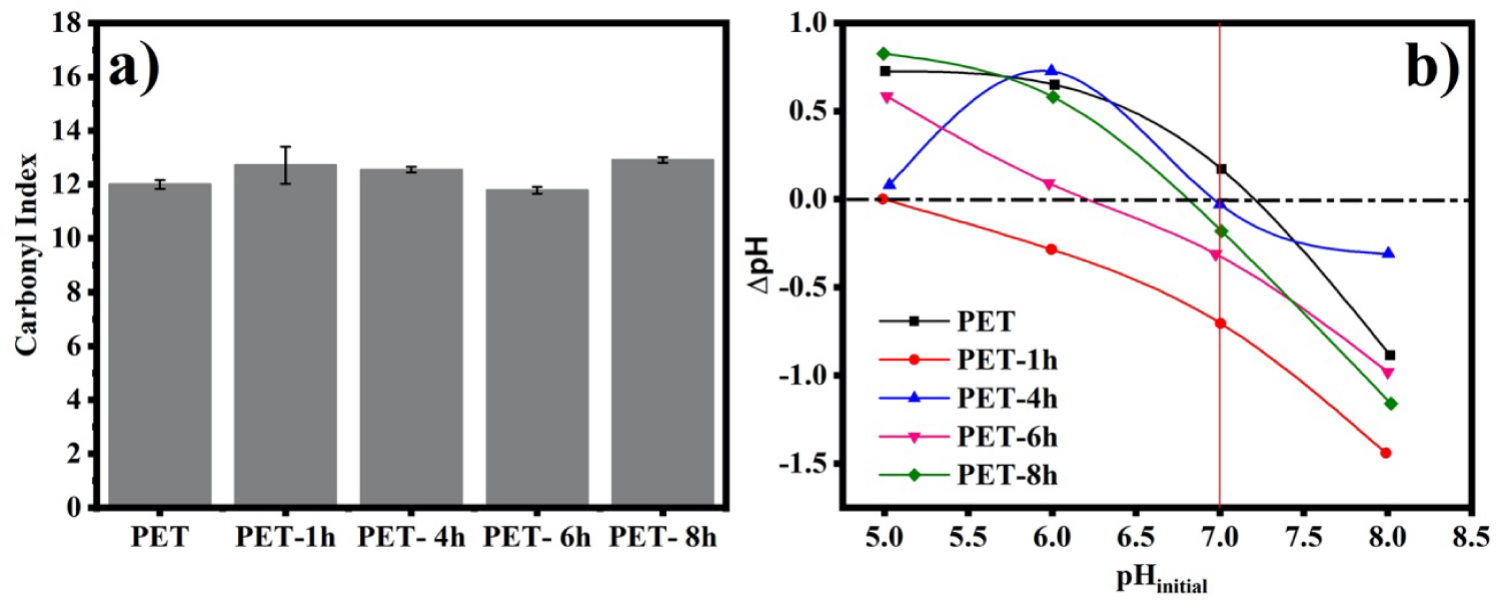
Carbonyl index of PET samples before and after undergoing accelerated aging (a) and pH_PZC_ values of microplastics before and after UV-B (b).

The carbonyl index, the ratio between C=O and CH_2_ stretching bands at 1720 and 2920 cm^–1^, is commonly used to assess plastic degradation.^18^ A slight increase in the carbonyl index was observed with weathering, rising from 12.00 to 12.91 for PET-8h and from 12.00 to 12.71 for PET-1h, while PET-6h showed a decrease to 11.80, likely due to the heterogeneous particle size affecting the oxidation rates. However, ANOVA revealed that only PET-8h exhibited a statistically significant difference compared to pristine PET (*p* ≤ 0.05) (Figure 2a), suggesting that the increase in carbonyl groups via the Norrish type I radical reaction was more pronounced in PET-8h.

Despite that, the pH_PZC_ results indicate a decrease in pH_PZC_ after the photodegradation process for all aged PETs, and values obtained were as follows: pristine PET (7.21), PET-1h (5.00), PET-4h (6.96), PET-6h (6.21), and PET-8h (6.81) (Figure 2b), probably due to the increase in δ- (−C=O) groups during aging, which is consistent with the results of Wang and collaborators.^35^

**Figure 3:**
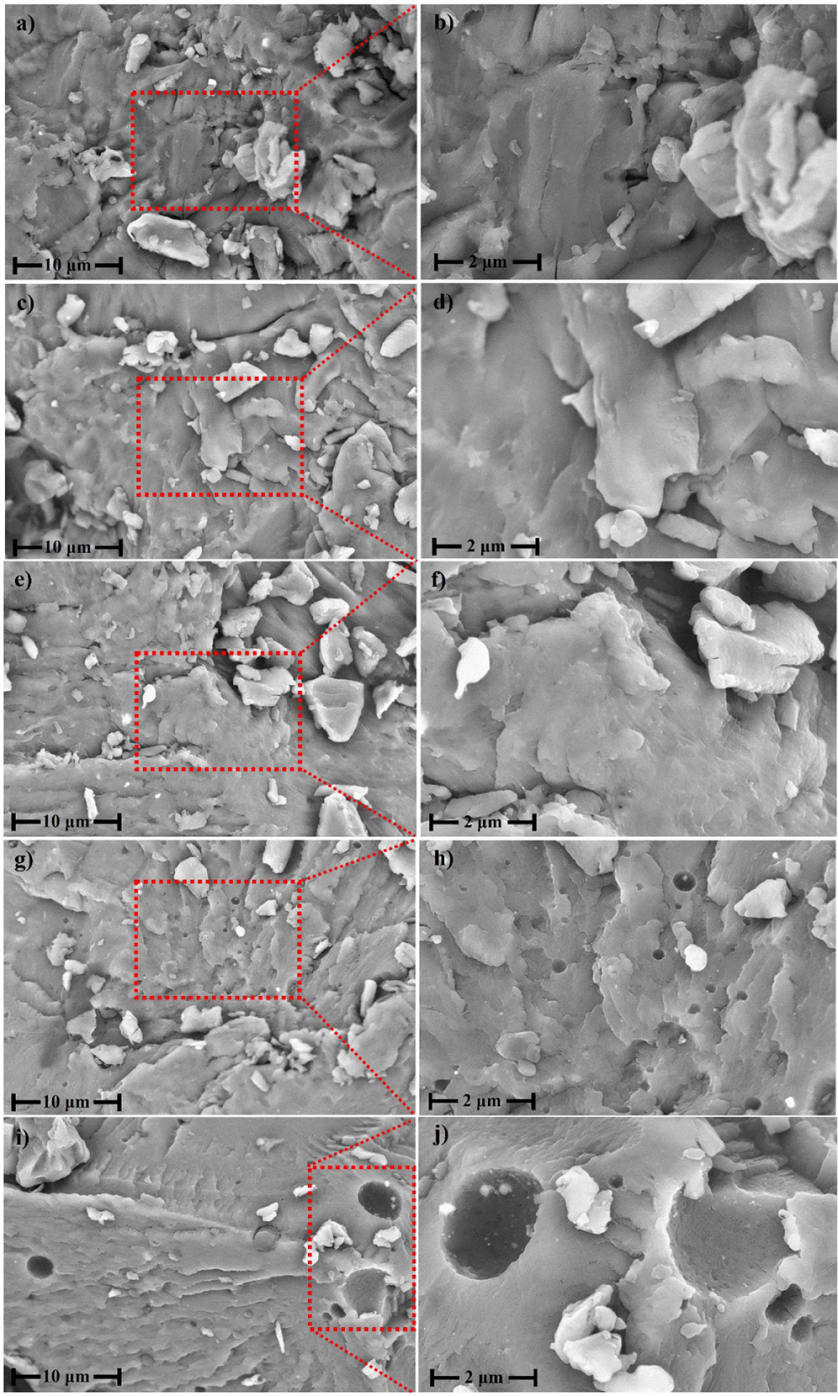
Scanning electron microscopy images (magnifications 2 and 5k) of PET (a, b); PET-1h (c, d), PET-4 h (e, f), PET-6 h (g, h), and PET-8 h (i, j).

**Figure 4:**
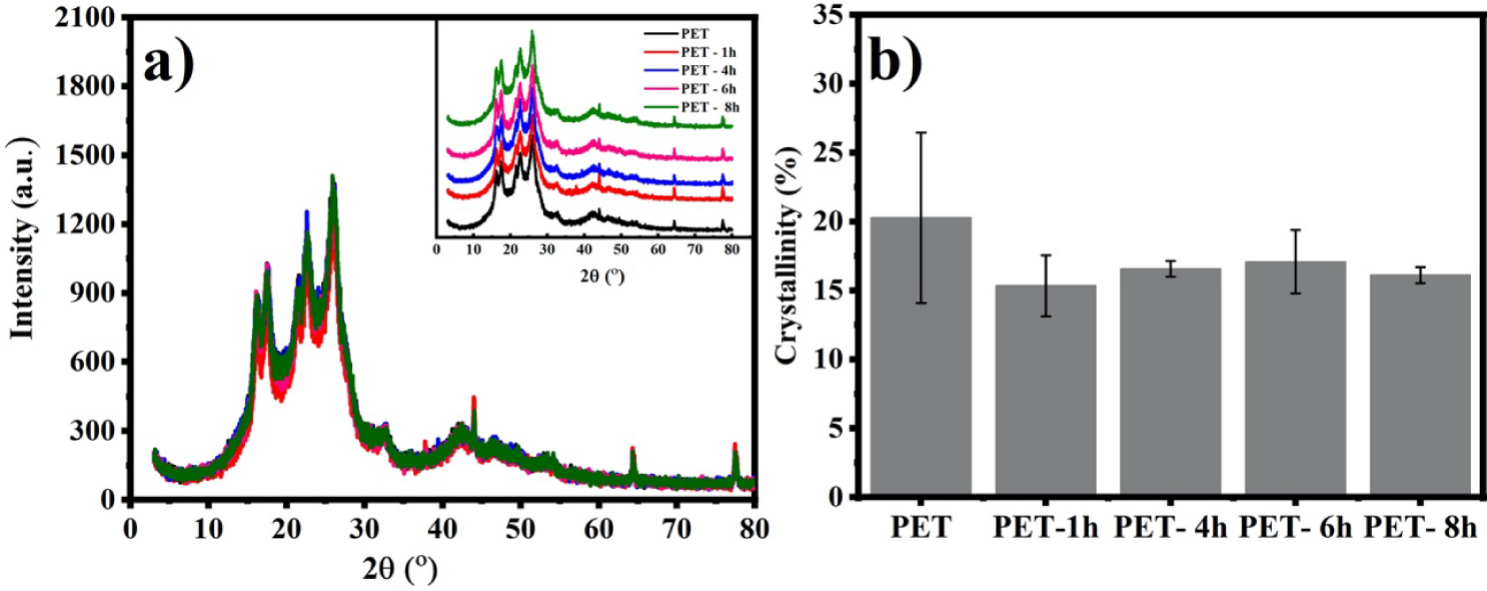
X-ray diffraction pattern of PET MPs (a) and degree of crystallinity (%) of microplastics before and after aging with UV-B radiation (b).

These findings are supported by the SEM micrographs (Figure 3), which reveal surface changes such as increased roughness and the formation of well-defined pores in samples aged for 6 h (Figure 3g,h) and 8 h (Figure 3i,j), indicating more advanced degradation.^36^ In contrast, microplastics aged for 1 h (Figure 3c,d) and 4 h (Figure 3e,f) exhibited no significant surface alterations, consistent with a minimal increase in carbonyl index. These surface modifications align with the oxidation trends indicated by the rising carbonyl index.

SEM analysis (Figure 3) revealed heterogeneous surfaces characterized by clusters of irregularly shaped particles, which are typical of amorphous or semicrystalline materials.^36^ PET can be either amorphous or semicrystalline depending on synthesis conditions, influencing its rigidity, impact resistance, thermal stability, and optical properties.^37^ X-ray diffractograms (Figure 4a) of both pristine and UV-B-aged PET show similar patterns, indicating the presence of both amorphous and crystalline phases. Diffraction bands, characteristic of semicrystalline polymers, display broad reflections from planes (010), (010), (110), and (100) at scattering angles 2θ = 17°, 22.5°, and 25.5°, with spacings of 5.21, 3.95, and 3.49 Å, respectively.^38,39^

Some studies reported a decrease in the PET percentage of crystallinity after photodegradation, which might suggest a structural change in the polymers after the photoaging process.^40,41^ However, in this study, the ANOVA results revealed no significant difference at the level of 5% for aged materials when compared to that of pristine PET. Thus, the accelerated photoaging process in a UV-B chamber used in this study did not alter the crystallinity of the aged materials (Figure 4b).

**Figure 5:**
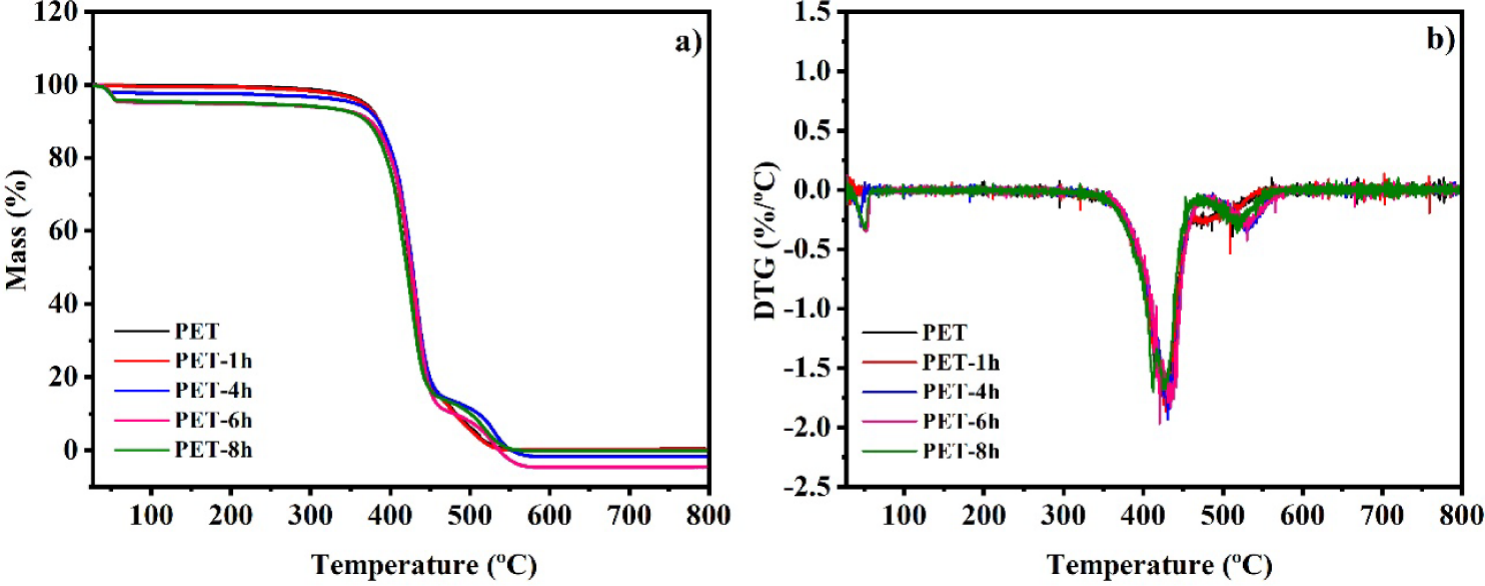
Thermograms (a) and DTG (b) acquired for microplastic samples before and after aging with UV-B.

The impact of the photodegradation process on the thermal stability of PET was evaluated using thermogravimetric analysis (TGA) (Figure 5a,b). The thermograms of pristine PET and PET-1h displayed similar behavior, with a single pyrolysis stage occurring at 427.17 and 429.23 °C (*t*_max_), respectively, and weight losses of 99.60% and 99.82%. This is likely due to the long PET chains breaking into shorter chains, which are subsequently depolymerized into low molecular weight volatile polymers.^35,42^

For PET samples exposed to UV-B for longer periods (PET-4h, PET-6h, and PET-8h), the pyrolysis process occurred in three stages. The first stage, observed at 43.22 °C, 50.87 °C, and 49.47 °C (*t*_max_) with weight losses of 2.12%, 4.66%, and 4.06%, respectively, likely corresponds to the volatilization of water. These results are consistent with the increase in the carbonyl index, indicating a rise in C=O groups, which enhances the material’s hydrophilicity. The second stage, occurring at 430.74 °C, 434.11 °C, and 426.71 °C (*t*_max_) with weight losses of 84.11%, 85.05%, and 81.95% for PET-4h, PET-6h, and PET-8h, respectively, is attributed to the breakdown of the long PET chains into shorter fragments. Notably, the weight loss percentage decreases with longer UV-B exposure as the photodegradation process progressively breaks the polymer chains. A thermal stage typically expected at 220–270 °C, corresponding to the pyrolysis of volatile residues and low molecular weight polymers^35,42^ was absent. Instead, a third stage was observed at 529.17 °C, 530.76 °C, and 518.96 °C (*t*_max_), with weight losses of 15.37%, 14.81%, and 13.81% for PET-4h, PET-6h, and PET-8h, respectively. This suggests that PET fragments released during UV-B degradation may have recombined, forming a new stable chemical structure, as proposed by Pires et al.^43^

Based on the SEM, carbonyl index, pH_PZC_, and TGA results, it is evident that UV-B exposure caused surface roughness in PET after 6 and 8 h, indicating increased degradation. The carbonyl index showed a rise in C=O groups, particularly after 8 h, consistent with oxidative processes, while TGA revealed that prolonged UV–B exposure led to multistage pyrolysis, reflecting the breakdown of long polymer chains and potential recombination of PET fragments into new, stable structures. Together, these findings highlight PET’s progressive structural and chemical changes with increased UV-B photodegradation exposure time.

**Figure 6:**
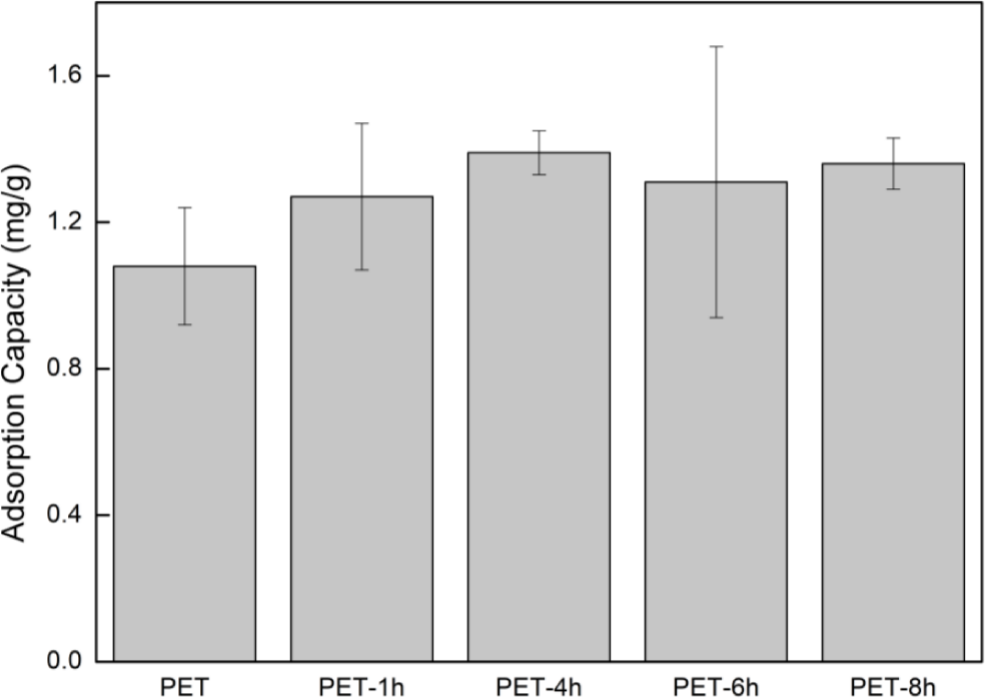
Equilibrium adsorption capacity of the MPs tested toward CP pesticide ([CP]_0_ = 1 mg L^–1^, MP size = 50–100 μm; pH_0_ = 7; *T* = 30 ^◦^C; [MP] = 0.2 mg mL^–1^.

An adsorption study was carried out to evaluate the effect of the photodegradation of PET on the adsorption capacity of microplastics concerning the organophosphate pesticide chlorpyrifos. The results are summarized in Figure 6 and suggest that the changes in physical, chemical, and morphological properties observed in the materials after photodegradation tests resulted in an increased adsorption capacity of the aged materials compared to the pristine ones.

The aged materials after 4 h (PET-4h and PET-8h) differ statistically at the 5% level regarding their performance in chlorpyrifos adsorption. The PETs adsorption capacity (mg g^–1^) followed the order: unaged PET (0.91 ± 0.17), PETs aged 1 h (1.10 ± 0.220), 4 h (1.24 ± 0.06), 6 h (1.15 ± 0.40), and 8 h (1.20 ± 0.09).

**Figure 7:**
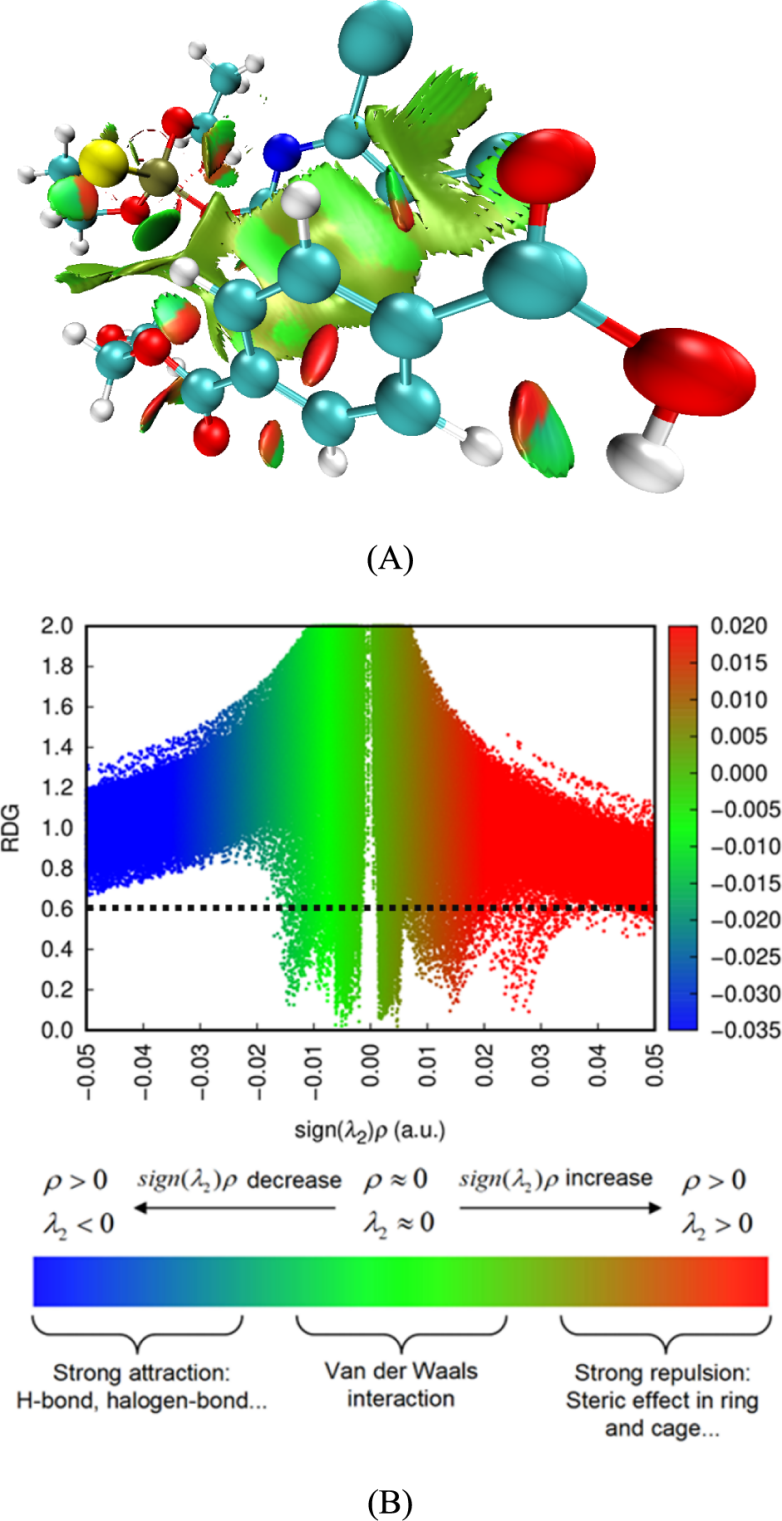
Noncovalent interactions (NCI) surface shown in the aged PET molecule over the chlorpyrifos molecule (A) and scatter plot (B) from DFT calculations.

According to the quantum chemical calculations, after the aging process, the PET molecule changes its geometry, since the C=O group involves an sp^2^ type bonding, the aged molecule becomes more planar compared to the nonaged PET molecule (Figure 3). This geometrical change also influences the adsorption of chlorpyrifos since many sp^2^ bonds electronically interact with the sp^2^ bonds of the aged PET polymer and, therefore, can increase the polymer’s adsorption capacity, which was confirmed by the NCI (Figure 7). At the same time, the benzene ring in PET can also generate π–π interactions with chlorpyrifos, indicating that the benzene ring was involved in the adsorption process, and aged PET might adsorb chlorpyrifos through π–π conjugation. This pattern of behavior was also identified in several adsorption studies that used microplastics, as documented by (9,35,44,45).

Figure 7 shows that the major interactions between aged PET and chlorpyrifos are indeed focused on aromatic rings between them, along with the sp^2^ bonding from aged PET interacting with chlorpyrifos, albeit there is some strong repulsion when compared to the van der Waals interactions that the calculation showed. In this context, the energy balance between these interactions favors the van der Waals interactions as there is experimental evidence of adsorption, which validates the van der Waals interaction surfaces.

## 4 Conclusion

The current study aimed to elucidate pesticide interaction with microplastics, specifically examining how photo-oxidation affects the adsorption of chlorpyrifos. PET was aged under varying UV-B exposure times. Characterization results indicated aging-induced surface deterioration, the generation of oxygen-containing functional groups, chain scission, and decreased hydrophobicity, particularly for microplastics exposed to UV-B radiation for longer durations (PET-6h and -8h). However, XRD and crystallinity (%) tests indicated that there was no change in the crystalline structure of the materials. Quantum chemical calculations showed that aged PET molecules changed their geometry and became more planar compared to nonaged PET. Adsorption test indicated an increase in adsorption capacity (mg g^–1^) for aged PETs: -4h (1.24 ± 0.06) and -8h (1.20 ± 0.09) compared to the unaged one (0.91 ± 0.17). These results have indicated the great potential of microplastics to adsorb micropollutants and potentially act as vectors in aquatic environments and that environmental factors such as photoaging affect the sorption of microplastics. This study establishes a theoretical and experimental foundation for understanding pristine and aged microplastics as carriers of hydrophobic pesticides and reveals the underlying adsorption mechanisms.

## Supporting Information Available

Figure S1: accelerated aging chamber and accelerated aging chamber equipped with UV-B fluorescent lamps; Figure S2: photographs of PET, PET-1h, PET- 4h, PET- 6 h, and PET-8h; Figure S3: Norrish type I mechanism applied for PET monomer, R^1^ and R^2^ are the alcohol and benzyl groups, respectively, P refers to the polymer molecule, and PH is the polymer molecule with a hydrogen atom; Figure S4: calculated UV–vis spectrum for PET monomer with intensity peaks above the spectrum plot and molecular scheme data; Table S1: one-way ANOVA results – carbonyl index; Table S2: student’s *t* test – carbonyl index (95% confidence interval); Table S3: one-way ANOVA results – degree of crystallinity; Table S4 – one-way ANOVA results – adsorption capacity; and Table S5 student’s *t* test – adsorption capacity (95% confidence interval) (PDF)
