# Supplementary material for: Impact of UV-B Photoaging on Chlorpyrifos Adsorption by PET Microplastics: Insights from Experimental and DFT Analysis
Source: ACS Omega. 2024 Nov 6;9(46):46439–46. doi: 10.1021/acsomega.4c07521 (PMC11579747; doi:10.1021/acsomega.4c07521)
Supplement: Supplementary file 1 — ao4c07521_si_001.pdf [file ao4c07521_si_001.pdf]

Supporting Information

Impact of UV-B Photoaging on Chlorpyrifos Adsorption by PET Microplastics: Insights from Experimental and DFT Analysis

Thais B. O. Costa[a]; Giuliana B. Santana[a]; Eric M. Silva[a]; Kelven G. A. Conceição[a]; Gabriela Z. Diaz[a]; Diego Q. Melo[b]; Antonia Mayza M. França[c]; Ronaldo F. do Nascimento[c], André G. Oliveira[d], Rílvia S. Santiago-Aguiar[e], Othon S. Campos[f], Carla B. Vidal[a,c*]

[a]Department of Chemistry and Biology, Federal University of Technology – Paraná, Deputado Heitor de Alencar Furtado St., Five Thousand Ecoville, Zip Code 81280-340 Curitiba, PR, Brazil.

[b]Institute of Education and Science of Sertão Pernambucano, PE 647, km 22, PISNC N-4, Campus Petrolina Zona Rural, Zip Code 56302-970 Petrolina-PE, Brazil.

[c]*(Actual)* Department of Analytical Chemistry and Physical Chemistry, Federal University of Ceará, Humberto Monte S/N Campus do Pici, Bloco 940, Zip Code 60451-970 Fortaleza, CE, Brazil.

[d]Center of Technological Sciences, University of Fortaleza, Av. Washington Soares, 1321, Edson Queiroz, Zip Code 60881-905 Fortaleza, CE, Brazil.

[e]Department of Chemical Engineering, Federal University of Ceará, Campus do Pici, Bloco 709, Zip Code 60455-760, Fortaleza, Ceará, Brazil

[f]Department of Physics and Chemistry, Federal University of Espirito Santo, Alto Universitário SN, Guararema, Zip Code 29500-000 Alegre, ES, Brazil.

*Corresponding author. E-mail: carlavidal@ufc.br

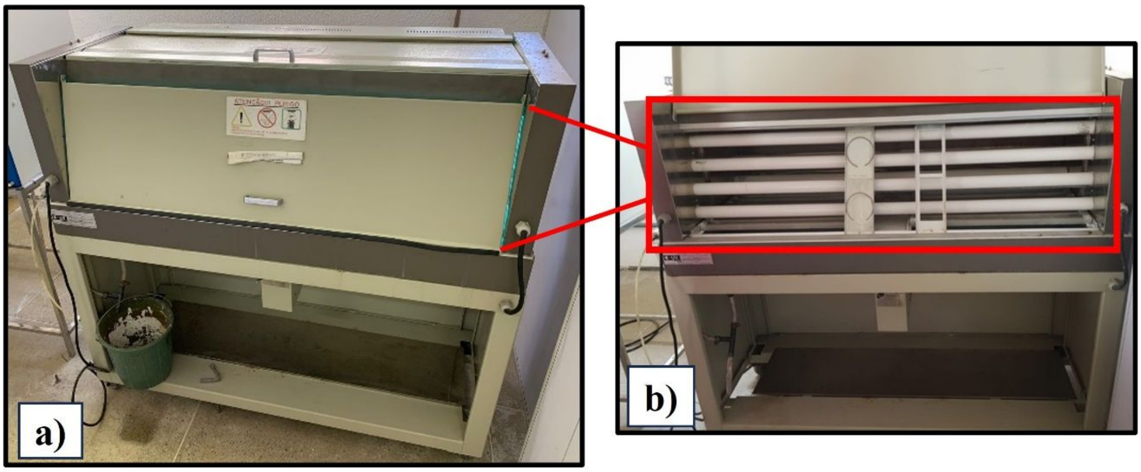

Figure S1- (a) Accelerated aging chamber and (b) Accelerated aging chamber equipped with UV-B fluorescent lamps.

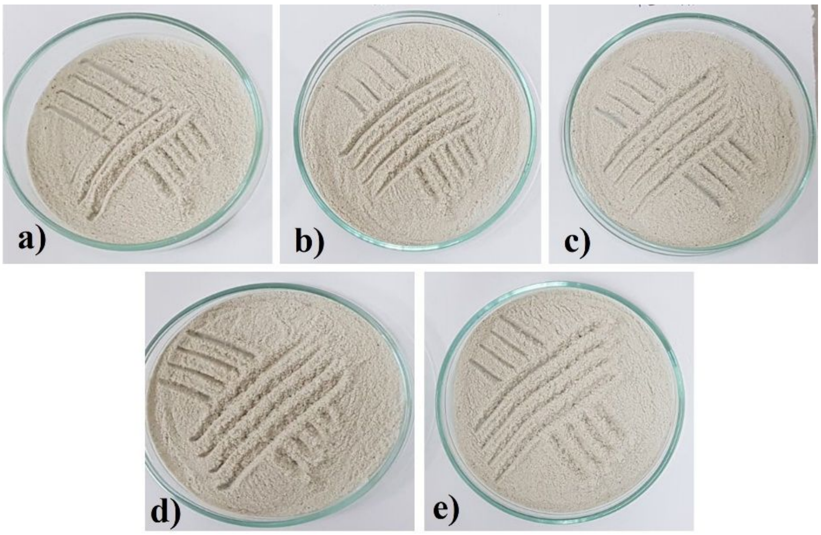

Figure S2 – Photographs of a) PET, b) PET-1h, c) PET- 4 h, d) PET- 6 h, and e) PET-8h.

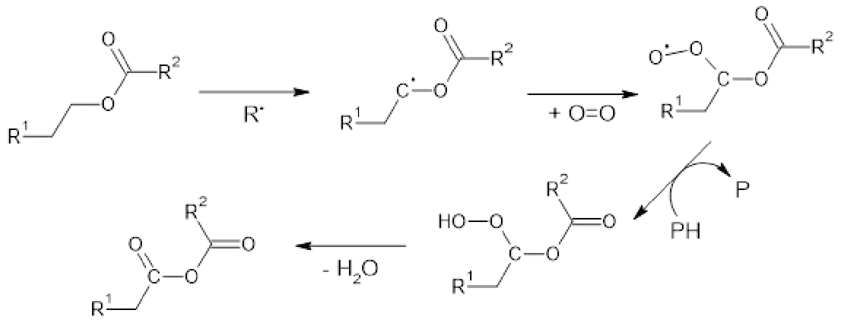

Figure S3 – Norrish type I mechanism applied for PET monomer. R¹ and R² are the alcohol and benzyl groups, respectively. P refers to the polymer molecule, and PH is the polymer molecule with a hydrogen atom.

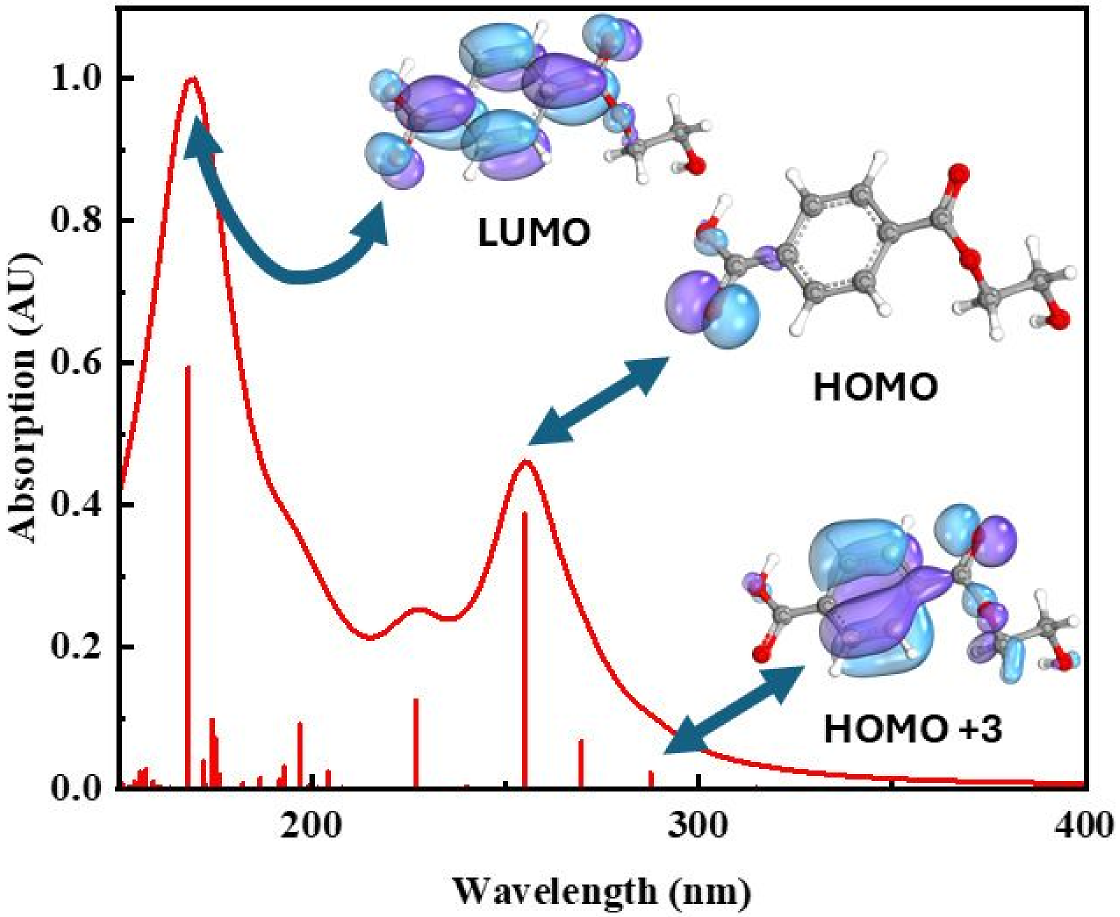

Figure S4 - Calculated UV-Vis spectrum for PET monomer with intensity peaks above the spectrum plot and Molecular scheme data.

Table S1 – One-way ANOVA results– Carbonyl index

**SUMMARY**				
Groups	Count	Sum	Average	Variance
PET	3	36.007	12.002	0.041
PET 1h	3	38.150	12.717	0.708
PET 4h	3	37.654	12.551	0.017
PET 6h	3	35.356	11.785	0.027
PET 8h	3	38.716	12.905	0.014

**ANOVA**						
Source of variation	SS	df	MS	f	p-value	F-crit
Between Groups	2.743	4	0.686	4.248	0.029	3.478
Within Groups	1.614	10	0.161			
Total	4.357	14				

As shown in Table S1, the P-value is 0.029, which is lower than the typical significance level (< 0.05), indicating that there is sufficient evidence to assert a significant difference between the means of the five tested material groups. The F-critical value (3.478) is lower than the F-value (4.248), which confirms a significant difference between the means. When the variation between groups is significantly greater than the variation within groups, as observed in the table, it suggests that at least one group mean differs from the others. Since a significant difference between groups was observed, Student's t-tests were conducted to compare the means of two specific groups (PET vs. PET 1h; PET vs. PET 4h; PET vs. PET 6h; PET vs. PET 8h) (Table S2).

Table S2 - Student's t-test - Carbonyl index (95% confidence interval).

	PET	PET 1h
Mean	12.002	12.717
Variance	0.041	0.708
Observations	3	3
Hypothesis of mean difference	0	
df	2	
Stat t	-1.430	
P(T<=t) one-tailed	0.145	
t crit one-tailed	2.920	

P(T<=t) two-tailed	0.289	
t crítico two-tailed	4.302	

	PET	PET 4h
Mean	12.002	12.551
Variance	0.041	0.017
Observations	3	3
Hypothesis of mean difference	0	
df	3	
Stat t	-3.947	
P(T<=t) one-tailed	0.015	
t crit one-tailed	2.353	
P(T<=t) two-tailed	0.029	
t crítico two-tailed	3.182	

	PET	PET 6h
Mean	12.002	11.785
Variance	0.041	0.027
Observations	3	3
Hypothesis of mean difference	0	
df	4	
Stat t	1.444	
P(T<=t) one-tailed	0.111	
t crit one-tailed	2.132	
P(T<=t) two-tailed	0.222	
t crítico two-tailed	2.776	

	PET	PET 8h
Mean	12.002	12.905
Variance	0.041	0.014
Observations	3	3
Hypothesis of mean difference	0	
df	3	
Stat t	-6.664	
P(T<=t) one-tailed	0.003	
t crit one-tailed	2.353	
P(T<=t) two-tailed	0.007	
t crítico two-tailed	3.182	

Table S3 – One-way ANOVA results– Degree of crystallinity

**SUMMARY**				
Groups	Count	Sum	Average	Variance
PET	3	60.810	20.270	57.230

PET 1h	3	46.000	15.333	7.363		
PET 4h	3	49.700	16.567	0.490		
PET 6h	3	51.250	17.083	7.957		
PET 8h	3	48.370	16.123	0.506		

**ANOVA**						
Source of variation	SS	df	MS	f	p-value	F-crit
Between Groups	43.217	4	10.804	0.734	0.589	3.478
Within Groups	147.091	10	14.709			
Total	190.308 1733	14				

Although the ANOVA did not identify a significant difference among the five groups as a whole, the Student's t-test can reveal differences in specific pairs. This may occur because ANOVA assesses the total variability among all groups, while the t-test focuses on individual comparisons. Thus, Student's t-tests were conducted, and the results confirmed that there is insufficient evidence to assert a significant difference between the means of the pairs (PET vs. PET 1h; PET vs. PET 4h; PET vs. PET 6h; PET vs. PET 8h).

Table S4 – One-way ANOVA results– Adsorption capacity

**SUMMARY**				
Groups	Count	Sum	Average	Variance
PET	3	3.917	1.306	0.019
PET 1h	3	4.427	1.476	0.032
PET 4h	3	4.737	1.579	0.003
PET 6h	3	4.535	1.512	0.105
PET 8h	3	4.663	1.555	0.004

**ANOVA**						
Source of variation	SS	df	MS	f	p-value	F-crit
Between Groups	0.140	4	0.035	1.077	0.418	3.478
Within Groups	0.325	10	0.032			
Total	0.464	14				

Based on the F-value and the p-value, we can conclude that there are no statistically significant differences among the five groups (PET, PET 1h, PET 4h, PET 6h, PET 8h) (Table S4). Although the ANOVA did not identify a significant difference among the five groups as a whole, the Student's t-test can reveal differences in specific pairs. This may occur because ANOVA assesses the total variability among all groups, while the t-test focuses on individual comparisons. Therefore, Student's t-tests were conducted, and the results can be viewed in Table S5.

Table S5 - Student's t-test - Adsorption Capacity (95% confidence interval).

	PET	PET 1h
Mean	1.306	1.476
Variance	0.019	0.032
Observations	3	3
Pooled variance	0.026	
Hypothesis of mean difference	0	
df	4	
Stat t	-1.301	
P(T<=t) one-tailed	0.132	
t crit one-tailed	2.132	
P(T<=t) two-tailed	0.263	
t crítico two-tailed	2.776	
	**PET**	**PET 4h**
Mean	1.306	1.579
Variance	0.019	0.003
Observations	3	3
Pooled variance	0.011	
Hypothesis of mean difference	0	
df	4	
Stat t	-3.236	
P(T<=t) one-tailed	0.016	
t crit one-tailed	2.132	
P(T<=t) two-tailed	0.032	
t crítico two-tailed	2.776	
	**PET**	**PET 6h**
Mean	1.306	1.512
Variance	0.019	0.105

Observations	3	3
Pooled variance	0.062	
Hypothesis of mean difference	0	
df	4	
Stat t	-1.013	
P(T<=t) one-tailed	0.184	
t crit one-tailed	2.132	
P(T<=t) two-tailed	0.368	
t crítico two-tailed	2.776	

	PET	PET 8h
Mean	1.306	1.554
Variance	0.019	0.003
Observations	3	3
Pooled variance	0.011	
Hypothesis of mean difference	0	
df	4	
Stat t	-2.883	
P(T<=t) one-tailed	0.022	
t crit one-tailed	2.132	
P(T<=t) two-tailed	0.045	
t crítico two-tailed	2.776	
